# Ethical Considerations for Enrolling “Invested Parties” in Large-Scale Clinical Studies: *Insights from the RECOVER Initiative*

**DOI:** 10.1002/eahr.500221

**Published:** 2024

**Authors:** Kellie Owens, Emily E. Anderson, Shari Esquenazi-Karonika, Keith Hanson, Maika Mitchell, Janelle Linton, Jasmine Briscoe, Leah Castro Baucom, Liza Fisher, Rebecca Letts, Kian Nguyen, Brendan Parent

**Affiliations:** division of medical ethics in the Department of Population Health at the New York University Grossman School of Medicine; Stritch School of Medicine at the Neiswanger Institute for Bioethics & Health Policy at Loyola University of Chicago; Department of Integrative Health at the New York University Grossman School of Medicine; University of Illinois College of Medicine at Peoria and the Order of Saint Francis Healthcare Children’s Hospital of Illinois; RECOVER Clinical Science Core Administration & Operations of the New York University Grossman School of Medicine; Department of Population Health at the New York University Grossman School of Medicine; Department of Population Health at the New York University Grossman School of Medicine; RECOVER Initiative representative; RECOVER Initiative representative; RECOVER Initiative representative; RECOVER Initiative representative; division of medical ethics in the Department of Population Health at the New York University Grossman School of Medicine

**Keywords:** human subjects research, research ethics, study recruitment, conflicts of interest, informed consent, privacy, self-experimentation

## Abstract

Research institutions often lack policies addressing the risks and benefits of enrolling “invested parties” such as investigators, research staff, and patient, caregiver, and community representatives (groups most affected by a disease or intervention) in studies where they have direct involvement. Invested parties may have both strong motivations to study the condition or intervention and to participate as study subjects. More guidance is needed to promote appropriate access to research participation and mitigate potential risks. This article addresses the gap in guidance by presenting an ethical framework and practical guidelines for the enrollment of invested parties. Drawing from experiences with the Researching COVID to Enhance Recovery (RECOVER) Initiative, a large multisite observational cohort study, we argue that invested parties should not be categorically excluded from enrollment in their own research studies if certain criteria are met and appropriate safeguards are in place. We underscore the need to balance inclusion with fairness, promote valid voluntary informed consent, ensure data privacy, protect scientific validity, and mitigate unique risks to invested parties as participants. Additionally, we recommend regular reporting and empirical assessment to evaluate the impact of enrolling invested parties on participants and study outcomes.

There has been little discussion in the bioethics literature about the risks of researchers enrolling in their own studies.^1^ Many research institutions have policies addressing the potential enrollment of employees in research studies, though these policies do not generally consider the involvement of parties who are directly involved in the design and implementation of the research itself. These parties include the principal and co-investigators of studies, research staff, and patient, caregiver, and community representatives (groups most affected by a disease or intervention), hereafter referred to as “invested parties.”

As research participants, invested parties might experience unique risks or unjust advantages that are not sufficiently addressed in regulatory requirements governing research with humans, conflict of interest policies, or the research ethics literature. Ethical concerns about enrolling invested parties are particularly salient in studies that seek to enroll large numbers of individuals and have broad inclusion/exclusion criteria because invested parties may not only have strong motivations to study the disease as researchers but also to participate in research studies of this type. Given the proliferation of large observational cohort studies such as the National Institutes of Health’s (NIH) All of Us Research program and other research biobanks and data repositories, more guidance is needed to promote appropriate access to research participation while also appropriately defining and mitigating potential risks to research participants.

Our interest in this specific issue arose from our involvement in the Researching COVID to Enhance Recovery (RECOVER) Initiative, a large-scale observational research study seeking to understand post-acute sequalae of SARS-CoV-2 infection or “long Covid.” All Americans have been affected in some way by Covid-19, and the RECOVER Initiative has broad inclusion/exclusion criteria. Enrollment is open to those who have had Covid-19 with and without post-acute sequalae, as well as those who have never had Covid-19. Given the number of sites nationally, many scientists, clinicians, research staff, and patient, caregiver, and community representatives may consider the opportunity to participate. For Covid-19 research, there may be good reasons to not only allow but to actively recruit health care workers due to their heightened risk of exposure and potential benefit from research participation.^2^ While the RECOVER Initiative is unique in some aspects, we believe it provides a good starting point from which to begin a more in-depth analysis of enrolling invested parties in research studies.

In this article, we describe some of the ethical concerns the RECOVER Initiative raised regarding enrollment of invested parties and how some study sites addressed these concerns. We examine the landscape of existing policies relevant to the enrollment of invested parties in clinical research. We apply Emanuel et al.’s framework for ethical clinical research to analyze unique considerations in enrolling different kinds of invested parties.^3^ We conclude with practical guidance for research leadership teams and institutional review boards (IRBs). Ultimately, we argue that the default position should be to allow invested parties to enroll in research if appropriate safeguards are in place, with some clear caveats.

## THE RECOVER INITIATIVE

Through the RECOVER Initiative, started in 2021, the NIH has been funding research on long Covid across the biomedical research spectrum, from basic science to large clinical studies. The Initiative aims to define the physiological changes that underlie long Covid, identify therapeutics to treat these changes, and develop measures to predict which patients will develop long-term effects from Covid-19.

The RECOVER Initiative brings together researchers from over 30 academic medical centers and health systems across the United States. In total, 198 participating study sites from 32 different regional hubs are involved. The research teams are collecting health data and biospecimens from both adults and children, including those who were and were not infected with SARS-CoV-2. By following participants over multiple years, the longitudinal studies will help characterize long Covid over time.

The ambitious scope of the RECOVER Initiative reflects the major threat posed by long Covid, which is estimated to impact between 10-30% of those infected with SARS-CoV-2. Defining the underlying biological causes of lingering symptoms and discovering effective treatments is a top priority for the NIH in order to reduce the burden of this post-viral syndrome on patients, health care systems, and society as a whole. The insights gained through the RECOVER Initiative will also enhance preparedness for future pandemics caused by novel pathogens.

A critical component of the RECOVER Initiative is incorporating input from patients, caregivers, and community members impacted by long Covid, hereafter referred to as Representatives. The RECOVER Initiative partners with over 70 Representatives and community engagement experts who are involved in various aspects of the research, providing insights on study design, recruitment and retention strategies, and effective communication of findings to community populations. Having the perspective of Representatives directly guide the science helps ensure that the priorities and experiences of those affected by post-acute sequelae are fully considered. By leveraging their lived experiences and expertise, this approach exemplifies how vital community participation is to patient-centered research on major public health challenges like long Covid. The RECOVER Initiative’s commitment to patient, caregiver, and community engagement and representation demonstrates the value of including the community’s voice at all stages of biomedical research.

As the RECOVER Initiative began recruitment and enrollment, those of us who were part of the ethics team, BP and KO, as well as another ethicist affiliated with a RECOVER Initiative site, EEA, began to field questions about the acceptability and mechanics of enrolling invested parties. We learned from communications with RECOVER Initiative staff that many sites had enrolled principal investigators and research staff members as participants, and that some but not all sites that had done so had special procedures in place to support enrollment of invested parties, including different payment arrangements, alternative consent processes or documentation, changes in data access, and revised study roles (e.g., in some cases staff did not engage with a protocol until they had completed it as a participant). We also learned that some local IRBs had prohibited the enrollment of invested parties. While we did not gather systematic data on enrollment of invested parties, we learned that such enrollment was already happening and was not uncommon. We also learned that there was no consensus on whether and what kinds of arrangements should be in place for invested parties to participate in the study.

The four vignettes below, based loosely on experiences from the RECOVER Initiative, describe the kinds of ethical challenges that may arise when invested parties enroll in research they have a hand in shaping.

### Vignette 1.

Study participants complete neurocognitive exams such as the Mini International Neuropsychiatric Interview, a brief, structured diagnostic interview for major psychiatric disorders, which asks highly personal and sensitive questions including some about illegal activities such as drug use. Study coordinators are trained to administer these interviews and know how they are scored. Study coordinators enrolled in the same study may feel uncomfortable administering or taking these exams with colleagues. When undergoing this testing as participants, study coordinators could be tempted to intentionally or inadvertently give the “right” answers or the answers their colleagues are looking for, thereby threatening the study’s scientific integrity. This raises questions about whether invested parties might have a preference (due to privacy risks) or even an obligation (due to risks to scientific integrity) to abstain from these tests, undergo these tests at another study site, or be disqualified from enrolling if these kinds of tests are a requirement of the study protocol. This also raises questions about whether enrollment of invested parties might also require modified informed consent procedures to ensure potential participants are aware that sensitive data about their health and behavior may be collected or accessed by their colleagues.

### Vignette 2.

As part of their study role, a physician investigator calls study participants to provide them with some of their study test results, answers any questions they may have, and recommends any needed follow up with their existing primary care or specialty physician. The physician investigator is less comfortable making recommendations to their colleagues. For example, a research coordinator participating in the study has an abnormal EKG that requires explanation and follow up, and the investigator feels conflicted about discussing this with their colleague who is also a casual friend. The physician investigator is concerned they may explain in more depth or make more firm recommendations because of their personal relationship with the research participant. Other study participants would likely be referred to their primary care physician to have a similar discussion. This situation raises concerns that an investigator may feel compelled to provide information to participants they know and work with that would not be provided to other study participants.^4^

### Vignette 3.

A research study coordinator is also a participant, as are their partner and several colleagues. The health clinic that employs the study coordinator and their colleagues has fairly strict policies regarding employees accessing their own or their family members’ medical records in the research process. This coordinator is worried about accidentally accessing records that they should not, including their own, since a large volume of data will be reviewed in the study and many people will have access to this information. They are concerned they might enter a medical record number to pull data, not realizing it is their own or a family member’s data, a concern that causes them significant anxiety while completing study tasks. At the same site, another investigator reviews the study documentation, including medical histories, and laboratory and other results. Because multiple study staff and other hospital employees are enrolled in the study, this investigator learns about their colleagues’ medical histories when reviewing documentation. This investigator is uncertain as to whether those colleagues are aware that the investigator has access to their information. Although study staff would know investigators will review their data, other hospital employees they work with may not (especially if this particular investigator is not named in the informed consent form). The investigator is particularly concerned about finding out sensitive information, such as a diagnosis of anxiety or depression. Both scenarios raise questions about whether additional oversight and communication about data privacy may be required to ethically enroll invested parties in research.

### Vignette 4.

A study investigator is also a participant and works at the hospital where study procedures and tests are done. Since this study investigator is not the principal investigator, the local IRB and hospital does not have a policy prohibiting them from participating in the study. As a result, the study investigator receives monetary compensation for participation in the study and time spent completing study tests. Since the study investigator lives outside the public transit coverage area for the city, they also receive a gas card to cover the cost of transportation. The investigator questions the appropriateness of these practices. Even though the study investigator is being treated the same as other study participants, they are being compensated for driving to their workplace, which they would do regardless of participating in the study, and study procedures are less of a burden for them compared to other study participants. This concern raises questions related to payment structures for the participation of invested parties and about which types of special arrangements may be appropriate for their participation.

## THE INSUFFICIENCIES OF EXISTING POLICIES

Federal regulations governing research with humans do not specifically address the recruitment and enrollment of employees in research nor the self-experimentation of investigators. While prior research has considered the ethical and policy issues regarding employees as research participants, there is little existing guidance regarding the enrollment of employees in research where they are directly involved as study personnel.^5^ The sparse existing guidance rarely addresses unique considerations that may pertain to those who design and lead the studies (e.g., principal investigators), those who are involved in recruitment, the informed consent process, data collection and analysis (e.g., study team members such as research nurses and biostatisticians), or those engaged in advisory or consulting roles (e.g., what the RECOVER study has called “Patient, Caregiver, and Community Representatives”).

Two types of institutional policies tangentially address the enrollment of invested parties in their own research: policies for the enrollment of employees, and policies for investigator self-experimentation. For employees, the main concerns discussed in prior literature are coercion and undue influence.^6^ For investigators, the main concerns include appropriate risk and benefit analysis and the accuracy of reported results.

Publicly available institutional policies that address the enrollment of employees and/or investigators vary in their requirements and are often vague. While some policies discourage the enrollment of employees, others allow employees to enroll in clinical research if several criteria are met.^7^ For example, participation cannot be a condition of employment, and extra measures to protect participant confidentiality may be required. There are few suggestions as to what form these measures might take. In some cases, IRBs must approve participation of employees for each study, or in the case of studies with greater than minimal risk.^8^ Some institutional policies specify that participants should initiate contact to enroll (that is, active recruitment is not allowed), while others allow more active methods of recruitment if they are not initiated by a direct supervisor. Institutional policies often compare employee participation in research with student participation due to similar concerns about coercion and undue influence, and some policies have a combined statement for both groups.^9^ Very few statements specifically refer to employees enrolling in studies they work on directly, with the exception of an NIH policy related to the enrollment of NIH staff members in studies within their own work unit.^10^

Policies on self-experimentation explicitly address the participation of principal investigators and co-investigators in studies they are directly involved in or running. Our review suggests that these kinds of policies are less common than policies addressing the enrollment of employees, although institutions may have standard practices not codified in publicly available policies. Some of the existing policies we found require IRB approval for enrollment of individual principal investigators or co-investigators into a study, while others prohibit self-experimentation.^11^ These policies aim to ensure that investigators have accurately accounted for the risks and benefits of enrolling, given their investment and presumed belief in the intervention. Some policies emphasize that investigators should meet inclusion and exclusion criteria for the study, and their enrollment and study procedures may be led by another staff member.^12^

There are some notable gaps in existing institutional policies and little discussion in the literature we are aware of regarding the enrollment of invested parties in research. Current policies address the enrollment of investigators or general employees of a health system, not other study personnel or Representatives. While institutions generally do not have policies specific to Representatives, a 2018 bioethics research initiative funded by the Patient-Centered Outcomes Research Institute did provide some guidance on enrolling Representatives in research.^13^ This guidance suggested that the ethical considerations for enrolling Representatives depended on their role in the study. The authors recommended that Representatives should not hold roles as both research participants and study personnel simultaneously but may be allowed to enroll if they serve as advisors on a research study, if proper precautions are in place. Despite this guidance, we have found little consensus on when the enrollment of invested parties may be appropriate in practice. Policies addressing investigators’ self-experimentation make little mention of potential conflicts of interest outside of ensuring the accuracy of study data from investigators. Policies on employee participation emphasize concerns about coercion and undue inducement but mostly ignore other important concerns such as risks to invested parties’ privacy, threats to data integrity, and fair subject selection. The inconsistency across policies suggests that policies are guided more by institutional risk management concerns rather than a well-considered ethical framework. Study features, such as inclusion/exclusion criteria and potential risks to participants (especially risk of disclosure of sensitive personal information), and institutional considerations, such as the availability of additional personnel to conduct certain study procedures, matter for determining appropriate enrollment of invested parties. Additionally, required safeguards may need to be altered depending on the type of invested party. For example, we may expect the primary safeguards for the enrollment of Representatives to look different from those for investigators because Representatives may have less control over study design or access to study data. A more nuanced framework is needed to guide decisions about enrollment of invested parties in research, one that identifies specific concerns and best practices for decision-making, and additional protections for each type of invested party in order to promote development of institutional guidance that is useful in practice.

## ETHICAL CONSIDERATIONS

Here we outline a framework to guide researcher and IRB decisions about including invested parties in research that aims to ensure that appropriate safeguards are in place to minimize potential harm while avoiding overprotection and unnecessary prohibition. We consider enrollment of invested parties in research in the context of the classic research ethics framework developed by Emanuel et al., which outlines seven criteria for ethical clinical research: social or scientific value, scientific validity, fair subject selection, favorable risk-benefit ratio, independent review, informed consent, and respect for potential and enrolled participants.^14^ Table 1 summarizes key ethical considerations (questions for investigators and IRBs to consider) and justification for inclusion as well as additional recommended safeguards.

### Social and scientific value.

For research to be ethical, it must be valuable. A key requirement of scientific value is generalizable results; research participants must be representative of a society for the results to be relevant. Categorical exclusion of any group, including invested parties, must therefore have reasonable justification due to sufficiently significant conflicts or concerns. This obligation drives our recommendation to allow invested parties to enroll in research with minimal exceptions.

### Scientific validity.

Enrolling invested parties in a research study could theoretically present some serious risks to the scientific validity of those studies if, for example, they do not appropriately meet inclusion/exclusion criteria or if their data are not accurate. However, these concerns are not unique to invested parties. Enrollment of individuals with certain access and/or authority may require special precautions but categorical exclusion is not ethically justified. These precautions may look different depending on the type of invested party. For example, investigators enrolled as participants in their own studies may have an incentive to falsify their records for their own privacy or to suit the aims of the study, as well as the power to manipulate their own and other study records. However, such incentives and power are present regardless of whether their own personal data is included. Additionally, as in vignette 1, study staff may have knowledge of study procedures that could affect their ability to objectively participate in parts of the study. Concerns about scientific validity may be less relevant for Representatives, whose control over study procedures is likely limited. While Representatives may have knowledge of study inclusion and exclusion criteria and could manipulate their ability to enroll in a study, we find this scenario to be unlikely and unworthy of their categorical exclusion, particularly in studies with broad inclusion criteria and appropriate screening procedures.

### Fair subject selection.

It would be unfair to universally exclude invested parties from research enrollment due to the potential benefits they could receive from participating. However, their enrollment should not come at the expense of underrepresented groups with limited access to research participation who could also derive benefits. Giving invested parties priority over others to enroll in a study would constitute unfair subject selection. A disproportionate number of participants from a specific type of group relative to their number in the population eligible to participate may not be a result of unfair subject selection; it could be a result of difficulty in getting people to participate for various reasons. Thus, concerns about fairness should not lead to a complete prohibition of participation by invested parties, but rather a default position of inclusion, ensuring that all relevant stakeholders have equitable opportunities to participate.

### Favorable risk-benefit ratio.

While prior bioethics analysis has focused on concerns related to undue inducement or even coercion, we argue that the more salient ethical concern for valid, voluntary informed consent of invested parties is that they may need, given their unique position, additional information or decision support to accurately assess the risks and benefits of their participation in research.^15^ Investigators and other study staff may be unduly confident in the benefits of the research or in the importance of the knowledge to be gained, potentially trivializing their own risks of participation. In this case, a research coordinator seeking informed consent from a senior investigator who wishes to participate in the study might feel either incapable of accurately assessing their superior’s risk comprehension or uncomfortable flagging their superior’s biased risk assessment. There could also be interpersonal risks that invested parties might not foresee prior to enrollment, such as when a colleague, supervisor, or supervisee is responsible for collecting information or performing tests that require intimate contact. Invested parties may not initially realize that coworkers might have access to sensitive personal data which they otherwise would not have. When possible, it might suffice to create degrees of separation. This could mean enrolling and managing an invested party at a different site or requiring staff who do not work directly with the invested party to obtain individuals’ consent to participate and manage sensitive data. In cases where this separation is not possible, it must be determined whether invested parties should be allowed to assume these risks with adequate disclosure during the informed consent process.

### Independent review.

The ethical requirement of independent review suggests that IRBs and data safety monitoring boards should be informed when investigators or anyone with power to shape the research design are enrolled in their own studies so that they can assess proposed changes to study design, methods, or inclusion/exclusion criteria with this information in mind. Relatedly, while we see no reason to preclude IRB members from enrolling in studies they have previously approved, their role in IRB review should be stopped upon enrollment. Senior investigators enrolled as participants should not have the authority to change the study design for their own benefit, e.g., by adding a test based on their own clinical picture, which is less relevant to the greater study population, or prioritizing the development of clinical trials focused on their own symptoms or demographics so they can match inclusion criteria. Consequential decisions about trial design should be made by multiple study team members to ensure that no single investigator can make decisions solely on their own self-interest.

### Informed consent.

As discussed above, policies related to enrolling employees in research mention concerns about potential undue inducement and coercion. Invested parties may feel compelled to participate if their research site is struggling with low enrollment, if their supervisors inflate the potential benefits of the study and/or minimize risks, or if they are worried about performance evaluations or advancement opportunities. However, these concerns should not require a blanket prohibition against enrolling invested parties in research; rather, additional safeguards may be needed for certain studies to ensure that participation will have no impact on employment status. To reduce the potential for coercion or undue influence, it may be appropriate to disallow direct recruitment measures for invested parties. More junior staff members might be assigned an independent support person, akin to a patient advocate, to assess the voluntariness of their enrollment decision. More likely is that study staff members may exhibit heightened enthusiasm for enrollment due to their dedication to the disease or phenomenon under investigation, their proximity to the study site, and their existing knowledge of the research objectives and purpose and thus require an informed consent process that highlights the unique risks that may arise due to their involvement in the study (e.g., privacy violations).

### Respect for potential/enrolled subjects.

Guidance is evolving regarding which results, including incidental findings, should and should not be disclosed to research participants, and the degree of control that individual participants should have over the decision to learn these results.^16^ Some invested parties might have access to their own study data that might not be available to other participants. This could include results of tests not verified by appropriately certified laboratories or genetic findings of uncertain clinical significance. Norms against disclosure of such results exist to protect research participants from relying or acting on unclear information to their own detriment, which applies equally to invested parties as to other participants. For invested parties who do not have access to their own data (such as Representatives), investigators may face complicated questions about their obligations to provide participants they know personally with study information that others do not receive. Prior research suggests that obtaining such information may cause significant concern for investigators, thus we recommend that researchers think in advance about whether this situation will arise, how they will handle it, and whether this type of situation will affect decisions to enroll invested parties.^17^ In some cases, deidentification of data, or tracking/auditing who accesses a participant’s study data might be sufficient to prevent invested parties from inappropriately accessing their own information or that of others they know personally. Clear protocols should be in place to standardize the way information is disclosed to participants, and special arrangements may be necessary to ensure that invested parties have appropriate contact with investigators regarding test results.

## PROCEDURAL STEPS FOR IMPLEMENTING SAFEGUARDS

Given our assessment of risks and benefits to potential research participants, threats to the social and scientific value and scientific validity of research, and impact on fair subject selection, to best respect and protect the interests of invested parties in research and to support valid, voluntary informed consent, we propose the following:

1. The default presumption should be that it is generally ethically acceptable to enroll invested parties in research.
2. Despite the default presumption, IRBs should provide explicit approval to enroll invested parties in research based on the principal investigator providing a satisfactory justification regarding consideration of the concerns in table 1 and implementation of appropriate safeguards as needed.
3. Narrow criteria provided in table 1 should be the basis for IRBs prohibiting enrollment of invested parties.
4. In the case of multisite studies, if enrollment at another local site is a possibility, this should be recommended over enrollment at one’s own site of employment.
5. Despite the default presumption of acceptability, any principal investigator may choose to categorically prohibit enrollment of invested parties and should justify and document this in the study protocol submitted for IRB approval. Reasonable justifications might include lack of resources or technical inability to provide appropriate privacy protections, a limited number of staff members to administer research procedures, or concerns about fair subject selection.
6. In only limited circumstances and with strong justification should IRBs be involved in determinations about the enrollment of a particular invested party; decisions about enrollment of invested parties should instead be made and applied equally for each category of invested party (principal investigators, coinvestigators, study staff/team members, and Representatives).
7. All invested parties enrolled in a study should be reported annually to the IRB and to the data safety and monitoring board, regardless of other reporting requirements.

## CONCLUSION

Our analysis suggests that in most large clinical research studies, an individual’s involvement as an investigator, staff member, or Representative on a research study should not automatically preclude them from participating. Because our analysis is based on experience with the RECOVER Initiative, we focus mostly on large observational research. We recognize that different types of research or study designs may have different ethical nuances that will require additional consideration. For example, while we expect that the ethical considerations and practical guidelines we provide are also applicable to studies that test an intervention, such as randomized clinical trials, there may be additional or heightened risks and benefits of enrollment to invested parties that are worthy of further consideration, such as a risk to scientific integrity if invested parties influence study design or procedures to access specific interventions for themselves. We would also expect that the risks related to enrolling invested parties, such as loss of scientific integrity or confidentiality, would be higher in smaller clinical behavioral or experimental studies than large-scale epidemiological studies. Additionally, our analysis focuses solely on ethical considerations related to enrollment of invested parties and does not address a range of other conflicts of interest that may be relevant to clinical research studies, including the disclosure and mitigation of competing financial interests.

We outlined above a range of criteria to guide consideration of special safeguards to promote voluntary participation, appropriate protections of confidentiality for personal health information, and assurance for scientific integrity. These considerations can be different for invested parties directly involved in research design (e.g., principal investigators), those with access to data and insider knowledge of study procedures (e.g., research staff) and those concerning staff with limited control over study design and procedures but knowledge of study goals and eligibility requirements (e.g., Representatives such as community advisory board members). Because Representatives may have different levels of access to study data or decision-making authority depending on the research design, safeguards for this role should be tailored to the specific needs of a given study.^18^ In multisite studies with high enrollment goals, some invested party enrollment safeguards might need to be established at the level of the clinical coordinating center, while others might best be left to individual study sites in coordination with their own IRBs that understand the culture and expectations of their own communities.

While previous research and institutional policies tend to focus on concerns of undue inducement and coercion, we argue that these risks can usually be effectively ameliorated by limiting active recruitment and providing unique supports for the informed consent process, such as information about who will have access to an individual’s private, de-identified data. This, however, raises a more significant ethical concern, that is, that investigators may find themselves in a position of knowing health information about a participant colleague that will not be shared with them. Thus, we recommend that investigators in an individual study consider whether this is a risk for their team and what, if anything, can be done to avoid potential conflicts of conscience.

Our analysis also suggests that one of the most valuable safeguards for the enrollment of invested parties in research is to require tracking and reporting of their enrollment and participation to IRBs (and data safety monitoring boards, if applicable). For multisite studies, this information should also be available to the lead principal investigator. Not only will this reporting provide important ethical oversight—it is the only way to empirically assess the prevalence and magnitude of the concerns we have detailed, such as harms to participants or breaches of study integrity.

## Figures and Tables

**Table 1.** Ethical Considerations for the Enrollment of “Invested Parties” in Clinical Research

Ethical requirement (Emanuel et al.)	Key ethical considerations	Justification for inclusion/additional recommended safe- guards
Social or scientific value	Would categorical exclusion of invested parties violate the requirement for representativeness and inclusiveness in subject selection?	If all other ethical requirements are sufficiently met, invested parties should be allowed to enroll.
Scientific validity	Could invested parties lie to be eligible for participation (or are all inclusion/exclusion criteria verifiable)? Could invested parties manipulate their own data?	A robust screening process and limiting access of invested parties enrolled in research to their own data should sufficiently mitigate against threats to scientific validity.
Fair subject selection	Are enrollment targets sufficiently large in proportion to the population of eligible individuals that invested parties do not have disproportionate access to the benefits of research?	If eligibility criteria are broad, invested parties should not be excluded. For reasons of equity and avoiding undue influence, invested parties should not be actively recruited for participation.
Favorable risk-benefit ratio	Are there any unique risks of research that might pertain to invested parties? For example, might colleagues or supervisors have access to sensitive personal information?	If unique risks are such that the risk/benefit balance is not acceptable for invested parties, they should be excluded. Otherwise, unique risks could be detailed in a consent addendum for invested parties.
Independent review	Are there individuals with singular authority over the study design, inclusion/exclusion criteria, recruitment methods, data management, or data analysis and reporting?	Any IRB member enrolled in a study should recuse themselves from IRB oversight of that study. No data safety and monitoring board member can be enrolled. In some instances, it may be desirable to exclude principal investigators of multisite studies from enrollment.
Informed consent	Will allowing the enrollment of invested parties promote undue inducement or coercion to participate? Do invested parties require additional information or decision support to fully understand the risks and benefits to their participation in the study?	In addition to prohibition of direct recruitment of invested parties, standard safeguards should be implemented to ensure valid, voluntary informed consent. Informed consent of any invested party should not be obtained by anyone in authority over that party. An IRB may recommend engagement of a third party (e.g. research subject advocate) during the informed consent process for all invested parties.
Respect for potential and enrolled subjects	Will the study generate any data that will not be shared with all research participants, such as tests conducted outside of certified laboratories? If so, can invested parties’ access to this information be adequately restricted? Will other study personnel have or require access to this information in a manner that identifies invested parties, potentially creating a conflict of conscience?	Depending on the tests being done and procedures around return of results, it may be reasonable or desirable to prohibit enrollment of all invested parties given the possibility of investigators and other team members to have access to certain results that will not be shared with participants. If enrollment of invested parties is determined to be acceptable, it may be reasonable or desirable to prohibit enrollment of biostatisticians, data managers, and others that have access to research-related results that will not be shared with participants, such as experimental laboratory or genetic tests, and to ensure that safeguards are in place to limit individuals from accessing their own and their colleagues’ identifiable data.
